# Study on the efficacy of compound porcine cerebroside and ganglioside injection in patients with ischemic stroke: A randomized, single‐center, open‐label, prospective study

**DOI:** 10.1002/ibra.12120

**Published:** 2023-07-15

**Authors:** Ya Chen, Xia Zhang, Hai‐Qing Zhang, Zhong Luo, Xue‐Jiao Zhou, Tao Liang, Fei Yang, Jun Zhang, Zu‐Cai Xu

**Affiliations:** ^1^ Department of Neurology Affiliated Hospital of Zunyi Medical University Zunyi Guizhou Province China

**Keywords:** compound porcine cerebroside and ganglioside injection (CPCGI), ischemic stroke, Modified Rankin Scale (mRS), National Institutes of Health Stroke Scale (NIHSS), neuroprotectant

## Abstract

Compound porcine perebroside and ganglioside injection (CPCGI) was used to treat stroke. The study was initiated because of the high incidence of low‐does CPCGI use in our area. However, no research has confirmed the effectiveness of CPCGI below the standard dose. Therefore, the aim of this study was to provide a reference for the clinical selection of different dose treatments. We collected ischemic stroke patients and divided them into three groups (low‐dose group: Group A = 4 mL, Group B = 6 mL, standard‐dose group: Group C = 10 mL). The modified Rankin Scale (mRS) scores, the National Institutes of Health Stroke Scale (NIHSS) scores, and the Barthel Index (BI) scores were performed before treatment and 14 days and 90 days post CPCGI treatment. For 90 days, the primary outcomes were calculated including the degree of disability, neurological recovery, and activities of daily living. All data were compared between pretherapy and posttreatment and among groups. NIHSS, mRS and BI scores improved on 14 and 90 days in each group. Group B and C improved than Group A on 14 and 90 days. The difference between groups B and C was not statistically significant. On 90 days, there were differences in the degree of disability, the recovery of neurological function, and the ability of daily living among groups. No drug‐related adverse reactions occurred in the groups. Although 4 or 6 mL CPCGI had some neuroprotective effects, the standard dose of 10 mL CPCGI had the best effect on reducing the degree of disability and improving abilities of daily living.

## Graphical abstract

Compound porcine perebroside and ganglioside injection (CPCGI) was used to treat ischemic stroke. However, there was no clinical evidence of low‐dose CPCGI. We conducted a clinical study to analyze whether the clinical results of low‐dose and standard‐dose CPCGI in patients with ischemic stroke were different. We collected ischemic stroke patients and divided them into three groups according to different therapeutic doses (Group A = 4 mL, Group B = 6 mL, Group C = 10 mL). The modified Rankin Scale (mRS) scores, the National Institutes of Health Stroke Scale (NIHSS) scores, and the Barthel Index (BI) scores were performed before treatment, 14 days, and 90 days post CPCGI treatment. During the 90 days, the primary outcomes were calculated including the degree of disability, neurological recovery, and activities of daily living. All data were compared between pretherapy and posttreatment and among groups.NIHSS, mRS, and BI scores improved on 14 days and 90 days in each group than pretreatment. Comparing the three scores among groups, Groups B and C improved significantly than in Group A on the 14 and 90 days. The difference between Groups B and C was not statistically significant. During the 90 days, there were differences in the degree of disability, the recovery of neurological functions, and the ability of daily living among groups. No drug‐related adverse reactions occurred in the groups. In the treatment of ischemic stroke, CPCGI may reduce the degree of disability and improve the abilities of daily living. Although low‐dose CPCGI had some neuroprotective effects, the standard dose had the best effect.

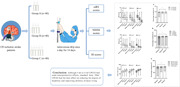

## 1 INTRODUCTION

Ischemic stroke is the most common type of stroke, accounting for nearly 70% of all strokes, and it is rapidly becoming a major public health challenge with high mortality and disability rate. Hence early prevention is the key; early diagnosis and treatment are also very important.^1^ Currently, the treatment of acute cerebral infarction includes (1) general management: blood pressure, blood glucose management, cranial pressure lowering, and complications management; and (2) specific treatment containing the improvement of cerebral circulation (intravenous thrombolysis, endovascular therapy, antiplatelet, anticoagulation, fibrin lowering, etc.), statin, and neuroprotection,^2^, ^3^, ^4^ among which thrombolysis and neuroprotection are two effective options for the treatment of acute cerebral infarction. However, thrombolysis is limited by the time window and many patients have missed the appropriate treatment period. Neuroprotective drugs can theoretically improve the prognosis of ischemic stroke, and animal studies have shown that neuroprotectants can improve the degree of neurological impairment.

Compound porcine perebroside and ganglioside injection (CPCGI) (Drug Approval No. H22026472; Jilin Buchang Pharmaceutical Co., Ltd) is approved by the Food Certification and Drug Administration of China. It is a sterile aqueous solution made by mixing healthy rabbit muscle extract and pig brain extract, containing 3.2 mg of peptide, 0.24 mg of ganglioside (GM1), and 0.125 mg of hypoxanthine per 1 mL, and so forth. CPCGI is a multitarget neuroprotective agent, which can improve cerebral blood circulation and cerebral metabolic function, accelerate the repair of damaged brain cells, reduce the damage of nerve tissue, and promote the normal physiological functions of diseased cells and hypoxic tissues.^5^, ^6^, ^7^, ^8^ Therefore, due to its multiple active ingredients, the drug may exert its neuroprotective effects by interfering with many different targets.^9^, ^10^, ^11^, ^12^ At present, more and more preclinical and clinical research studies are exploring the role and possible mechanisms of CPCGI in various neurological diseases.

Preclinical studies have shown that CPCGI can promote the expression of Beclin1 and PINK1, and inhibit the expression of Parkin in rats with cerebral ischemia/reperfusion injury, thus inhibiting neuronal apoptosis.^13^ Previous studies have shown that CPCGI has neuroprotective effects on middle cerebral artery occlusion rats by inhibiting apoptosis and improving synaptic and mitochondrial functions.^14^ In a rat trauma model, CPCGI was found to activate the Nrf2 signaling pathway and attenuate oxidative stress‐mediated calpain activation to produce a protective effect.^13^ In clinical studies, it was found to increase neuronal cell regeneration in acute craniocerebral injury, which can effectively improve the efficacy of craniocerebral injury and neurological injury, and its mechanism of action may be related to its downregulation of nerve peptide Y and S100β levels and upregulation of glial fibrillary acidic protein levels.^15^ Recent studies have shown that early treatment with CPCGI can effectively improve the symptoms of neurological deficits, improve the quality of life of patients, significantly reduce the level of serum of inflammatory factors, and protect the ischemic brain cells.^16^ In China, the drug is used for the treatment of stroke and other nervous diseases, requiring 10–20 mL to be added to 250 mL of 0.9% sodium chloride injection for intravenous infusion. The relatively high‐priced drug especially for poor areas in western China, causes the clinical phenomenon of using small doses for treatment. However, no research has confirmed the effectiveness of CPCGI below the standard dose. Therefore, the aim of this study was to evaluate the efficacy and safety of different doses of CPCGI in the treatment of ischemic stroke and to provide a reference for the clinical selection of different dose treatments.

## 2 METHODS

### 2.1 Study design and subjects

This was a prospective, single‐center, open‐label, randomized controlled study. The patients who were admitted to the Affiliated Hospital of Zunyi Medical University were consecutively included from December 2018 to September 2020. Patients who met the following inclusion criteria were eligible for the study: (1) meeting the diagnostic criteria for cerebral infarction, with onset over 24 h to within 2 weeks; (2) confirmed by brain computed tomography (CT) or magnetic resonance imaging (MRI); (3) age at onset of 18–75 years, regardless of gender; (4) 3 ≤ the National Institutes of Health Stroke Scale (NIHSS) ≤ 16; (5) not treated with neuroprotective drugs after the onset of stroke. Patients were excluded if they met one of the following exclusion criteria: (1) Any disability related to the central nervous system (CNS) that predated the stroke; (2) With a premorbid modified Rankin Scale (mRS) score of more than two and lived dependently. (3) Previously diagnosed cerebrovascular disease; (4) Confirmed intracranial occupying lesions, trauma, cerebral parasitism, and various cardiogenic cerebral embolisms including atrial fibrillation; (5) Ganglioside deposits; (6) Thrombolytic or peptic ulcer patients who cannot take aspirin; (7) Alanine aminotransferase (ALT), aspartate aminotransferase (AST) exceeding 1.5 times the upper limit of normality; urea nitrogen (BUN) exceeding 1.2 times the upper limit of normality; (8) Alcohol dependent, or drug dependent on other sedatives; (9) Pregnant and breastfeeding; and (10) Patients with impaired consciousness.

A total of 120 patients were randomly divided into Groups A, B, and C by using a computer‐generated random sequence (1:1:1). On the day of successful admission screening, patients were randomized. To ensure allocation is balanced within three groups, randomization was stratified. Sequence generation was performed using a computer‐generated randomized block design (with a block size of 6 or 9). Allocation concealment was ensured by the fact. The project researcher was responsible for implementing and delivering the results for each randomization to the care team by e‐mail.^17^ Patients in Groups A, B, and C were, respectively, given 4, 6, and 10 mL CPCGI, in an intravenous drip of 250 mL 0.9% sodium chloride once daily for 14 days.

**Figure 1:**
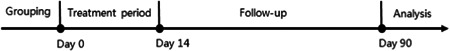
Diagram showing the flow of participants. The corresponding dose CPCGI of each group was diluted with 250 mL 0.9% sodium chloride injection as a solution and infused intravenously once a day until 14 days in the treatment period, at each visit point (Day 0, Day 14, and Day 90) to complete routine, blood biochemistry, and other laboratory tests; The mRS scores, NIHSS scores, and Barthel Index scores were performed; adverse reactions were observed and recorded. mRS, Modified Rankin Scale; NIHSS, National Institutes of Health Stroke Scale.

The trial protocol was shown in Figure 1. All patients were treated with basic pharmaceutical therapy consisting of Bay aspirin enteric‐coated tablets (Drug Approval No.J20171021, Bayer Medical and Health Co., Ltd) 100 mg orally 1/day and atorvastatin calcium tablets (Drug Approval No.H20163270, Lepu Pharmaceutical Technology Co., Ltd) 20 mg orally 1/day while allowing treatment for risk factors such as hypertension and diabetes, dehydrating agents to reduce brain cell edema, anti‐infection to prevent complications, as well as rehabilitation. Antihypertensive therapy in patients with early acute cerebral infarction (less than 72 h) whose blood pressure was less than 220/120 mmHg and who had no complications requiring emergency antihypertensive therapy was not required. The purpose of antihypertensive drugs for patients over 72 h after acute cerebral infarction was to lower blood pressure below 140/90 mmHg and hypoglycemic drugs were aimed to make the average plasma glucose 8.6 mmol/L.^18^, ^19^ In addition, antifibrinogen and other neuroprotective drugs were rejected.

The study was conducted according to the Declaration of Helsinki and was approved by the Ethics Committee of the Affiliated Hospital of Zunyi Medical University (Ethics Trial No. 77), China, on December 24, 2018; informed consent was obtained from patients or their families if the patient lost the capacity to give informed consent before they were enrolled, for using the patient's data for research.

### 2.2 Data collection

Demographic data, initial stroke severity assessed by baseline NIHSS scores, Modified Rankin Scale (mRS) scores and Barthel Scale (BI) scores, baseline systolic and diastolic blood pressure, blood routine examination and chemistries on admission, vascular risk factors, brain imaging, and stroke‐related complications during hospitalization were collected. The mRS scores, NIHSS scores, and BI scores were performed before treatment and 14 and 90 days after treatment, and the related indexes were monitored.

### 2.3 Clinical efficacy evaluation

Basic information, medical history and mRS scores, NIHSS scores, and BI scores at three visits were recorded for all patients. Patients were followed up on the 90 days by outpatient visits or telephone interviews. The primary outcomes^1^, ^20^ were the mRS scores, NIHSS scores,^21^, ^22^ and BI scores^23^ at 90 days after treatment. Based on the mRS score, 0–1 were asymptomatic or had no significant disability, 2–3 were mild/moderate disability, and 4–6 were severe disability or death. Based on the pre‐ and posttreatment NIHSS score, the degrees of recovery were classified as follows: (1) cured: 91%–100% reduction, (2) significantly improved: 46%–90% reduction, (3) improved: 18%–45% reduction, (4) no change: ≤17% increase or decrease, deterioration >18% increase. Based on BI score, categorized as (1) 100, independence; (2) 75–95, mild dependence; (3) 50–70, moderate dependence; (4) 25–45, severe dependence; (5) 0–20, complete dependence.^24^

The safety of medication was observed accurately. Blood routine, urine routine, stool routine, liver and kidney function, and other biochemical tests were detected during the three visits. Adverse reactions were recorded and treated in time. Patients with adverse reactions were followed up until the adverse reactions disappeared.

### 2.4 Statistical analysis

IBM SPSS Statistics 19 software was used for statistical analysis, in consideration of only individual subjects dropped out of our clinical trial and this experiment used the per‐protocol set (PPS) for statistical analysis. Continuous variables were expressed as mean, standard deviation, median, and quartiles; analysis of variance (ANOVA) and nonparametric tests were used according to the type of data, respectively; for categorical variables, data were expressed as the number of cases or composition ratios, and Pearson's *χ* ^2^ was used for analysis. The Kruskal–Wallis Test was used for the comparison of mRS scores, NIHSS scores, BI scores, and stratified comparisons between different groups, and the Mann–Whitney test was used for the comparison of mRS scores, NIHSS scores, and BIbefore and after treatment. Paired *t* test was used for differences in blood pressure before and after treatment in the three groups, and Fisher's exact test was used for differences in adverse reactions. *p* < 0.05 was considered a statistically significant difference.

There is no relevant research data to calculate the sample size. So, the purpose of this study was preliminary to observe and explore the efficacy and safety of small doses of CPCGI. Refer to the Regulation on Drug Registration approved by the US Food and Drug Administration (promulgated in 2005), and refer to the related literature.^25^, ^26^, ^27^ Based on the minimum sample size requirement of 20–30 patients in each group and about 20% possibility of shedding in clinical trials, a total of 120 patients were included.

## 3 RESULTS

### 3.1 Subjects recruitment, trial completion, and baseline characteristics of ischemic stroke in groups

**Figure 2:**
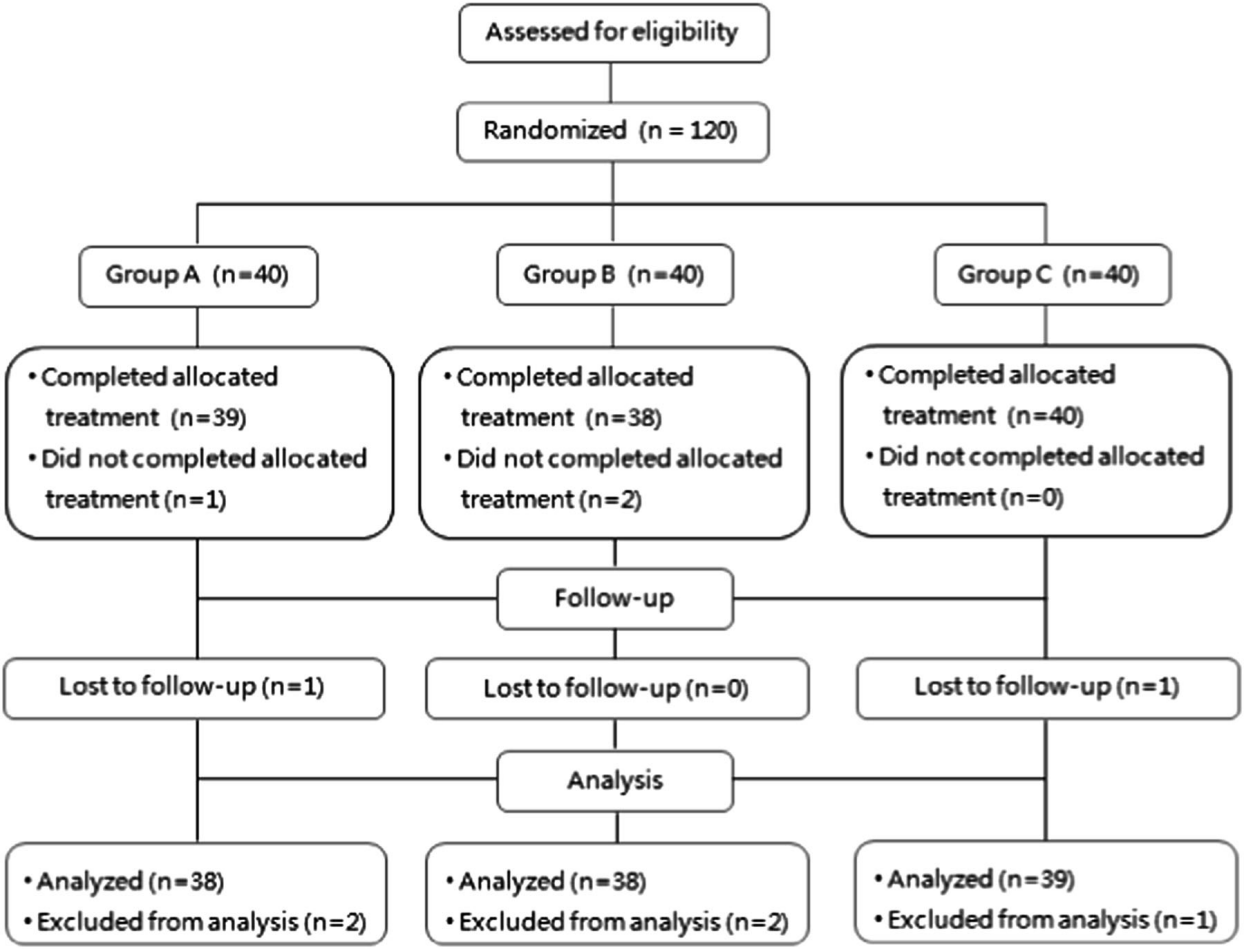
The general overview of trial interruption and completion.

A total of 120 subjects were recruited in the trial, respectively, 40 in Groups A, B, and C, and the male–female ratio was 29:11, 27:13, and 32:8. Figure 2 showed the overall trial disenrollment, which was 4.17% (5/120), and the reason was that the patients or their families refused to continue the trial, resulting in not following standard treatment or interrupting follow‐up. The final completion rates were 95% (38/40), 95% (38/40), and 97.5% (39/40) in Groups A, B, and C, respectively, with no statistical difference between the three groups (*p* > 0.05). The details of the baseline indicators of the three groups are shown in Table 1, with no statistical difference between the groups (*p* > 0.05).

**Table 1:** Comparison of baseline indicators in subjects with ischemic stroke between the three groups.

Baseline	Group A	Group B	Group C	Statistical values	*p* Values
Age	60.70 ± 11.26	61.05 ± 12.30	64.51 ± 11.81	*F* = 1.255	0.289
Gender				*χ* ^2^ = 1.619	0.445
Male	29 (72.5)	27 (67.5)	32 (80)		
Female	11 (27.5)	13 (32.5)	8 (20)		
Lipid metabolism disorders				*χ* ^2^ = 1.39	0.50
Yes	31 (77.5)	35 (87.5)	33 (82.5)		
No	9 (22.5)	5 (12.5)	7 (17.5)		
Hypertension				*χ* ^2^ = 1.25	0.54
Yes	25 (62.5)	29 (72.5)	29 (72.5)		
No	15 (37.5)	11 (27.5)	11 (27.5)		
Diabetes				*χ* ^2^ = 6.04	0.19
Yes	16 (40)	10 (25)	18 (45)		
No	24 (60)	30 (75)	21 (55)		
Heart disease				*χ* ^2^ = 0.75	0.69
Yes	12 (30)	9 (22.5)	12 (30)		
No	28 (70)	31 (77.5)	28 (70)		
Smoking				*χ* ^2^ = 1.43	0.49
Yes	27 (67.5)	24 (60)	29 (72.5)		
No	13 (32.5)	16 (40)	11 (27.5)		
Time to onset of symptom (d)	5.80 ± 4.24	5.18 ± 3.95	5.08 ± 3.06	*F* = 0.4256	0.654
BI sores	72.5 (60,80)	75 (58.75,80)	70 (65.0,85)	*χ* ^2^ = 0.317	0.853
NIHSS scores	5 (3,7)	4.5 (3,7)	5 (3,6)	*χ* ^2^ = 0.021	0.99
mRS scores	2 (2,4)	2 (2,3)	2 (2,3)	*χ* ^2^ = 0.563	0.755

*Note*: Age and time of onset are expressed as mean ± SD; gender and complications are expressed as number of cases (percentage); BI, NIHSS, mRS are expressed as median (interquartile spacing). ANOVA, *χ* ^2^ test, and Kruskal–Wallis test were used.

Abbreviations: BI, Barthel Index; mRS, modified Rankin scale; NIHSS, National Institutes of Health Stroke Scale.

### 3.2 Analysis of the effectiveness of CPCGI in patients with ischemic stroke

#### 3.2.1 mRS scores

**Figure 3:**
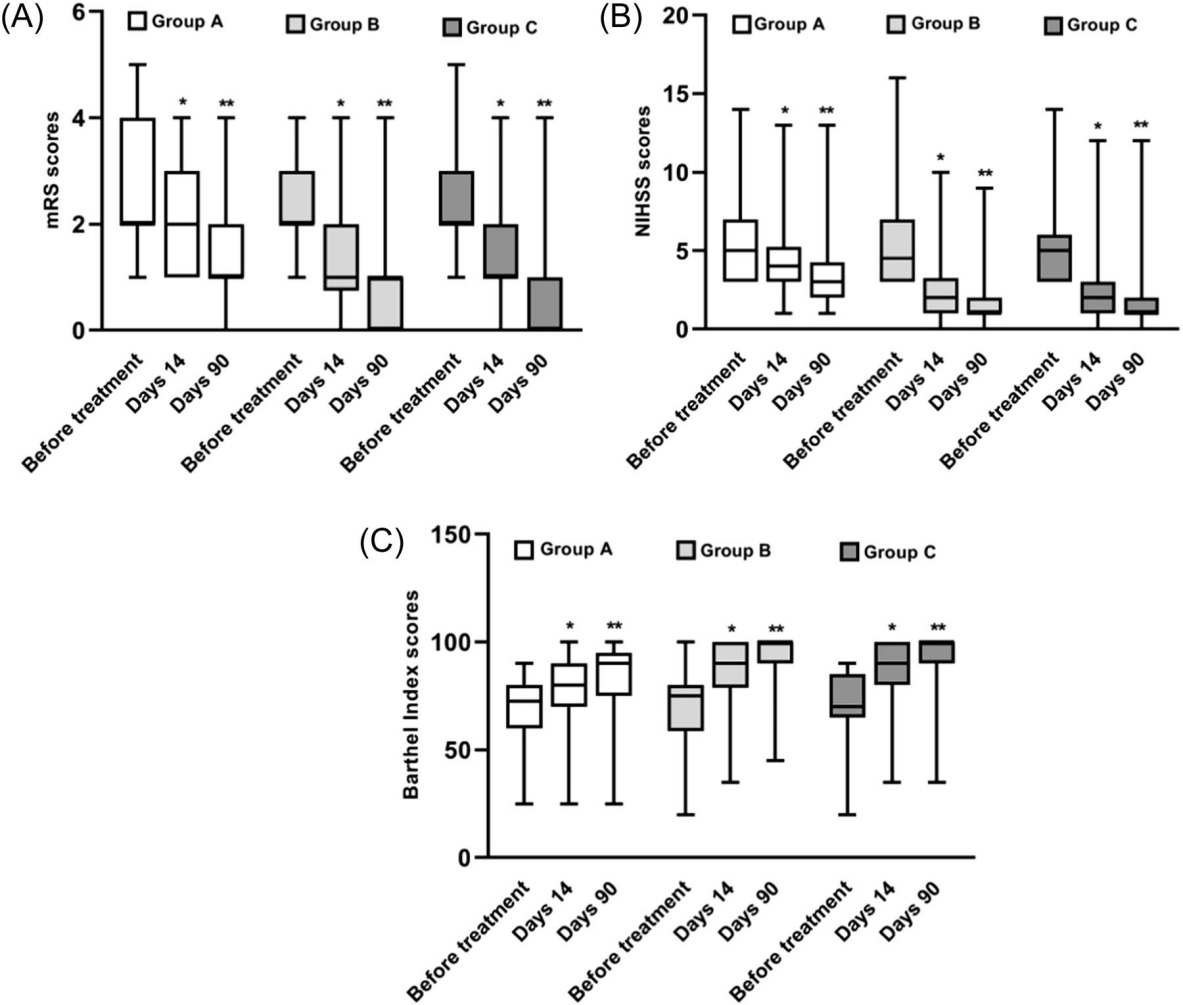
Comparison of mRS scores (A), NIHSS scores (B), and BI scores (C) before, 14 days, and 90 days after treatment with different doses of CPCGI. *p ＜ 0.05 between 14 days after treatment and before. **p ＜ 0.05 between 90 days after treatment and before. BI, Barthel Index; mRS, modified Rankin scale; NIHSS, National Institutes of Health Stroke Scale.

**Figure 4:**
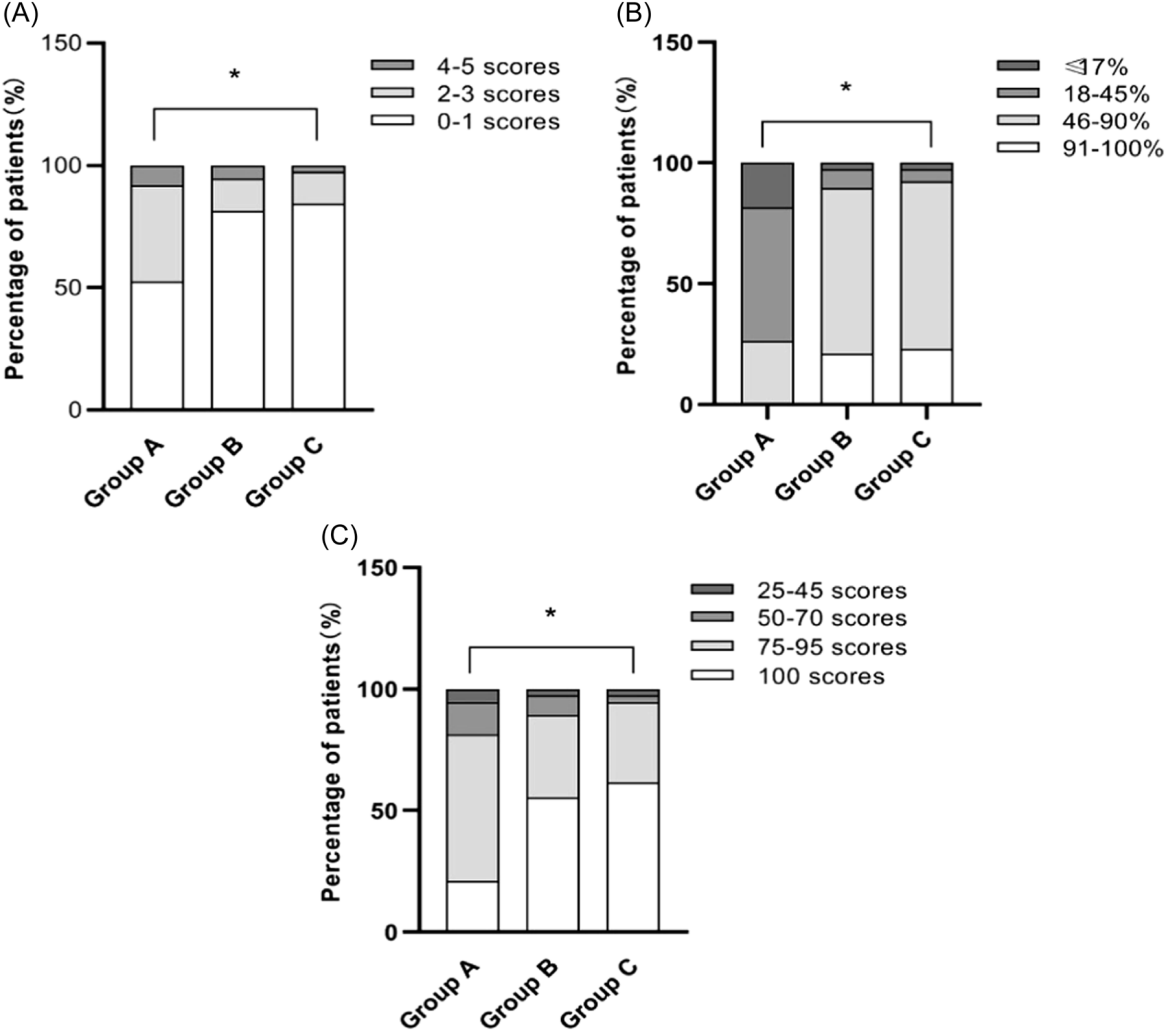
Comparison of efficacy assessment of different doses of CPCGI on day 90 after treatment. (A) Stratified by the size of mRS scores, the proportion of no disability (0–1 score): Group C > Group B > Group A (*p < 0.05); (B) Stratified by the proportion of decrease in NIHSS scores, the proportion of cured (91%–100% decrease in NIHSS scores): Group C > Group B > Group A (*p < 0.05); (C) Stratified by the size of BI, the proportion of complete independence (100 scores) in life activities: Group C > Group B > Group A (*p < 0.05). BI, Barthel Index; mRS, modified Rankin Scale; NIHSS, National Institutes of Health Stroke Scale.

The mRS scores in Groups A, B, and C improved at 14 and 90 days after administration compared with those before (*p* < 0.05), as shown in Figure 3. The results showed that all three doses could ameliorate disability. Therefore, to verify whether the curative effects of different dosage groups are different, we made a comparison between groups. Compared with Group A, the mRS scores in Groups B and C decreased more at 14 days and 90 days (*p* < 0.05), and the difference in mRS scores between Groups B and C was not statistically significant (*p* > 0.05), as shown in Table 2. After stratifying according to the size of the mRS scores at 90 days, the difference was statistically significant (*p* < 0.05) among Groups C, B, and A. In contrast, the rate of disability in Group C was the lowest than in Group B and Group A on Day 90 after treatment, which can be seen in Table 3 and Figure 4.

**Table 2:** Comparison of mRS scores, NIHSS scores, and Barthel Index by time point in the three groups for ischemic stroke treated with CPCGI.

	Group A	Group B	Group C	*χ* ^2^ Value	*p* Value
*mRS scores*					
Before treatment	2 (2, 4)	2 (2, 3)	2 (2, 3)	0.563	0.755
Day 14	2 (1, 3)	1 (0.75, 2)*	1 (1, 2)**	11.419	0.003
Day 90	1 (1, 2)	1 (0, 1)*	0 (0, 1)**	22.767	0.001
*NIHSS scores*					
Before treatment	5 (3, 7)	4.5 (3, 7)	5 (3, 6)	0.021	0.99
Day 14	4 (3, 5.25)	2 (1, 3.25)*	2 (1, 3)**	20.009	0.001
Day 90	3 (2, 4.25)	1 (1, 2)*	1 (1, 2)**	32.823	0.001
*BI scores*					
Before treatment	72.5 (60, 80)	75 (58.75, 80)	70 (65, 85)	0.371	0.853
Day 14	80 (70, 90)	90 (78.75, 100)*	90 (80, 100)**	12.725	0.002
Day 90	90 (75, 95)	100 (90, 100)*	100 (90, 100)**	17.97	＜0.001

*Note*: Data is expressed as medians (interquartile spacing) and was statistically analyzed using the Kruskal–Wallis test.

Abbreviations: BI, Barthel Index; mRS, Modified Rankin Scale; NIHSS, National Institutes of Health Stroke Scale.

* *p* < 0.05 between Groups A and B

** *p* < 0.01 between Groups A and C.

**Table 3:** Comparison of efficacy assessment at Day 90 after treatment with different doses of CPCGI.

	Group A (%)	Group B (%)	Group C (%)	*χ* ^2^ Value	*p* Value
mRS				11.699	0.011
0～1	20 (52.6)	31 (81.6)	33 (84.6)		
2～3	15 (39.5)	5 (13.2)	5 (12.8)		
4～5	3 (7.9)	2 (5.3)	1 (2.6)		
NIHSS reduction rate (%)				51.813	＜0.001
<17	7 (18.4)	1 (2.6)	1 (2.6)		
18–45	21 (55.3)	3 (7.9)	2 (5.1)		
46–90	10 (26.3)	26 (68.4)	27 (69.2)		
91–100	0 (0.0)	8 (21.1)	9 (23.1)		
BI				15.994	0.005
25–45	2 (5.3)	1 (2.6)	1 (2.6)		
50–70	5 (13.2)	3 (7.9)	1 (2.6)		
75–95	23 (60.5)	13 (34.2)	13 (33.3)		
100	8 (21.1)	21 (55.3)	24 (61.5)		

*Note*: Data are expressed as the sample size (composition ratio) and statistically analyzed using Pearson's *χ* ^2^ or Fisher's exact test.

Abbreviations: BI, Barthel Index; mRS, Modified Rankin Scale; NIHSS, National Institutes of Health Stroke Scale.

#### 3.2.2 NIHSS scores

NIHSS scores decreased in Groups A, B, and C at 14 and 90 days after medication compared with those before (*p* < 0.05), shown in Figure 3. The comparison was made among the three groups; NIHSS scores decreased more in Groups B and C than in Group A at 14 and 90 days (*p* < 0.001), but the difference between Groups B and C was not statistically significant (*p* > 0.05) as shown in Table 2. At 90 days, the percentages decline in NIHSS scores showed statistically significant differences among Group C, Group B, and Group A (*p* < 0.001), as shown in Table 3 and Figure 4. Although the results showed that all three doses could improve neurological disorders, the higher the dose, the greater the advantage.

#### 3.2.3 BI scores

BI scores were higher in Groups A, B, and C on the 14 and 90 days after medication compared with those before (*p* < 0.05); see Figure 3. In comparison among groups, BI was higher in Groups B and C than in Group A on the 14 and 90 days (*p* < 0.05), and the difference between Groups B and C was not statistically significant (*p* > 0.05), as shown in Table 2; stratified by the size of BI at 90 days, the highest independence of activity for life was found in Group C, successively Group B and Group A, with statistically significant differences (*p* < 0.05), as shown in Table 3 and Figure 4.

### 3.3 Safety analysis of CPCGI in patients with ischemic stroke

#### 3.3.1 Analysis of vital signs and laboratory tests

There was no difference in body temperature, respiration, and pulse before and after treatment (*p* > 0.05), but blood pressure was significantly lower after treatment than before in each group (*p* < 0.05). Laboratory tests including blood routine, electrolyte, liver and kidney function, myocardial enzymes, and blood coagulation function have no difference between the groups (*p* > 0.05), as shown in Table 4.

**Table 4:** Occurrence of adverse reactions in patients with ischemic stroke at different doses of CCPGI.

	Group A	Group B	Group C
*Systolic blood pressure (mmHg)*			
Before treatment	160.10 ± 22.31	160.00 ± 20.30	157.77 ± 19.35
On days 90 after treatment	133.63 ± 8.72	132.37 ± 7.11	134.31 ± 7.18
Statistical values (*t*)	8.69	8.64	8.24
*p* Value	0.000	0.000	0.000
*Diastolic blood pressure (mmHg)*			
Before treatment	92.50 ± 16.94	93.45 ± 13.79	91.31 ± 14.78
On days 90 after treatment	78.32 ± 12.27	76.82 ± 12.79	75.64 ± 9.92
Statistical values (*t*)	4.165	9.601	6.967
*p* Value	0.000	0.000	0.000
*Adverse reactions*			
Constipation	10	12	9
Respiratory tract infection	2	3	5
Fever	2	0	1
Dizziness	9	12	10
Headache	6	4	7
Abnormal blood count	3	1	2
Urine abnormalities	2	3	2
Positive fecal occult blood	3	2	1
Abnormal blood biochemistry	3	3	2
Serious adverse reactions	0	0	0
Drug‐related adverse events	0	0	0

*Note*: Blood pressure was expressed as the mean ± SD, Adverse reactions are expressed as the number of cases. Paired *t* test was used for differences in blood pressure before and after treatment in the three groups, and Fisher's exact test was used for differences in adverse reactions between groups.

#### 3.3.2 Adverse reactions

Adverse reactions occurred in Groups A, B, and C, shown in Table 4. There was no statistical difference in the occurrence of adverse reactions in each group (*p* > 0.05) and there were no drug‐related adverse reactions. Serious adverse reactions, which caused the suspension of the study, did not appear in all groups.

## 4 DISCUSSION

This study aimed to obtain clinical evidence of different doses of CPCGI in the treatment of ischemic stroke and to further evaluate its effectiveness and safety in the treatment of ischemic stroke. This study showed that the NIHSS scores and mRS scores decreased in Groups A, B, and C at 14 and 90 days after treatment with the corresponding doses of CPCGI, with a decreasing trend; while the BI increased compared to the pretreatment level, with an increasing trend. This provides some theoretical support for the clinical use of CPCGI, and the drug has a long‐lasting neuroprotective effect. Furthermore, to investigate which dose is the best, we conducted a comparison between groups, finding that compared with Group A, the NIHSS score and mRS score of patients in Groups B and C were significantly decreased, and the BI was significantly increased. There was no statistical difference between Group B and Group C, indicating that the efficacy of the 6 and 10 mL CPCGI was the best. Meanwhile, the efficacy of 6 mL CPCGI may be comparable to the standard dose of 10 mL. Obviously, from the trend of the data of each group, the improvement of each index was more obvious in Group C, but there was no difference in Group B, which may be related to the small statistical sample size.

Taking 90 days as the endpoint to evaluate the time point, the above indexes were used to further evaluate the treatment effect. In Group C, the proportion of mRS scores in the range of 0–1 was the highest, that is, the disability‐free rate was the highest, the proportion of NIHSS score (an index of neurological rehabilitation) decreased the most, and the proportion of complete independence after the illness was the highest, followed by the 6 mL group and 4 mL group.

In terms of safety analysis, no adverse effects related to CPCGI were found, but it was observed that both systolic and diastolic blood pressure decreased in patients after administration compared to the predose period. It can be attributed to the cerebrovascular autoregulation of cerebral vessels and the stress response of the body. Previous hypertension history, compensatory hypertension caused by occupying effects and stress reactions such as pain and urinary retention may all affect the blood pressure changes in the acute stage.^28^, ^29^, ^30^ As the recovery of the disease, blood pressure decreased, and antihypertensive drugs were used at the same time, so the systolic and diastolic blood pressure decreased before and after the drug was used, so this phenomenon has nothing to do with the use of CPCGI. The above results indicate that 2 weeks of CPCGI combined with basic medication may contribute to the recovery of neurological deficit in patients with ischemic stroke, reduce disability, and improve the daily living ability of patients.

However, our studies have limitations: firstly, small sample size; secondly, short follow‐up time; and thirdly, lack of blank controls. In addition, the NIHSS scores of the subjects enrolled in our study were mostly <10. Although there is no significant difference in baseline indicators before treatment (*p* ＞ 0.05), the symptoms of stroke patients may spontaneously relieve, especially slight disability, which may lead to the evaluation deviation of the results. Stroke comorbidities may be associated with the main outcome, such as anxiety, depression, obesity, bedsore, infection, and so forth, but there was no correlation analysis in the study. We will further confirm the neuroprotective effects of CPCGI by increasing the blank control, increasing the sample size, and increasing the follow‐up time, and find the most suitable effective therapeutic dose by increasing the number of different dosage subgroups, and explore the mechanism of CPCGI in patients with ischemic stroke at a deeper level, so as to provide a targeted treatment basis for the treatment of ischemic stroke.

## 5 CONCLUSION

These findings indicated that 2 weeks of treatment of CPCGI combined with basic pharmaceutical therapy may be conducive to recovery by reducing disability rates and neurological damage and improving patients' activities of daily living in patients with ischemic stroke. Meanwhile, the curative effect might be preserved for 90 days or even longer. Although 4 or 6 mL CPCGI may be a safe and effective treatment for patients with ischemic stroke, 10 mL CPCGI showed the best curative effect. The study confirms some effectiveness and safety of doses (4, 6 mL) of CPCGI in the treatment of ischemic stroke and provides a reference for the clinical selection of different dosing treatments. However, as far as the best result is concerned, 10 mL CPCGI standard dose therapy should achieve the best clinical effect when applied to stroke patients. Nevertheless, there are still some limitations in this study, and we intend to further study some clinical problems of CPCGI, such as the administration time, safety and effectiveness when the dose is greater than 10 mL, and even the neuroprotective mechanism.

## AUTHOR CONTRIBUTIONS

Ya Chen, Jun Zhang, and Zu‐Cai Xu conceived and designed the experiment. Ya Chen, Xia Zhang, Xue‐Jiao Zhou, Hai‐Qing Zhang, Tao Liang, Zhong Luo, and Fei Yang acquired the data while Ya Chen and Zu‐Cai Xu analyzed them. All authors critically revised the manuscript for important intellectual content and approved the final manuscript. Ya Chen and Zu‐Cai Xu are the study guarantors.

## CONFLICT OF INTEREST STATEMENT

The authors declare that this study received funding from Jilin Buchang Pharmaceutical Co. Ltd. The funder was not involved in study design, data collection and analysis, decision to publish, or preparation of the manuscript. The authors declare that there are no other conflicts of interest. [Correction added on 2 September 2025, after first online publication: In this version, the conflict of interest statement was updated at the request of the author.]

## ETHICS STATEMENT

All the participants signed the informed consent to participate in the study. Informed consent was obtained from patients or their families if the patient lost the capacity to give informed consent before they were enrolled, for using the patient's data for research. The present study was performed in accordance with the Declaration of Helsinki, which is a statement of ethical principles that directs physicians and other participants in medical research involving human subjects. The participants were assured about the anonymity and confidentiality of their information. Moreover, the study was approved by the Ethical Committee of the Affiliated Hospital of Zunyi Medical University (Ethics Trial No. 77). The trial was registered in the Chinese Clinical Trial Registry (ChiCTR Number: ChiCTR2500096309). [Correction added on 2 September 2025, after first online publication: In this version, the ethics statement was updated at the request of the author.]

## Data Availability

Due to the need to ensure the confidentiality policies of participants and national laws, the data sets generated and/or analyzed in the current research are not public but can be obtained from the corresponding author according to reasonable requirements.
